# Preliminary Radiogenomic Evidence for the Prediction of Metastasis and Chemotherapy Response in Pediatric Patients with Osteosarcoma Using ^18^F-FDG PET/CT, *EZRIN*, and *KI67*

**DOI:** 10.3390/cancers13112671

**Published:** 2021-05-28

**Authors:** Byung-Chul Kim, Jingyu Kim, Kangsan Kim, Byung Hyun Byun, Ilhan Lim, Chang-Bae Kong, Won Seok Song, Jae-Soo Koh, Sang-Keun Woo

**Affiliations:** 1 Department of Nuclear Medicine, Korea Institute of Radiological and Medical Sciences, Seoul 01812, Korea; xikian@kirams.re.kr (B.-C.K.); nmbbh@kirams.re.kr (B.H.B.); ilhan@kirams.re.kr (I.L.); 2 Radiological & Medico-Oncological Sciences, University of Science & Technology, Seoul 34113, Korea; jingyu8754@kirams.re.kr; 3 Division of Applied RI, Korea Institute of Radiological and Medical Science, Seoul 01812, Korea; krmount@kirams.re.kr; 4 Department of Orthopaedic Surgery, Seoul National University Hospital, Seoul 03080, Korea; cbkongmd@gmail.com (C.-B.K.); wssongmd@gmail.com (W.S.S.); 5 Department of Pathology, Korea Institute of Radiological and Medical Sciences, Seoul 01812, Korea; jskoh@kirams.re.kr

**Keywords:** *KI67*, *EZRIN*, ^18^F-FDG PET/CT, random forest, radiogenomics, chemotherapy response, metastasis

## Abstract

**Simple Summary:**

Pediatric osteosarcoma is one of the most aggressive cancers, and predictions of metastasis and chemotherapy response have a significant impact on pediatric patient survival. Radiogenomics, as methods of analyzing gene expression or image texture features, have previously been used for the diagnosis of chemotherapy responses and metastasis and can reveal the current state of cancer. In this study, we aimed to generate a predictive model using gene expression and ^18^F-FDG PET/CT image texture features in pediatric osteosarcoma in relation to metastasis and chemotherapy response. A predictive model using radiogenomics technology that incorporates both imaging features and gene expression can accurately predict metastasis and chemotherapy responses to improve patient outcomes.

**Abstract:**

Chemotherapy response and metastasis prediction play important roles in the treatment of pediatric osteosarcoma, which is prone to metastasis and has a high mortality rate. This study aimed to estimate the prediction model using gene expression and image texture features. 18F-fluorodeoxyglucose positron emission tomography/computed tomography (^18^F-FDG PET/CT) images of 52 pediatric osteosarcoma patients were used to estimate the machine learning algorithm. An appropriate algorithm was selected by estimating the machine learning accuracy. ^18^F-FDG PET/CT images of 21 patients were selected for prediction model development based on simultaneous *KI67* and *EZRIN* expression. The prediction model for chemotherapy response and metastasis was estimated using area under the curve (AUC) maximum image texture features (AUC_max) and gene expression. The machine learning algorithm with the highest test accuracy in chemotherapy response and metastasis was selected using the random forest algorithm. The chemotherapy response and metastasis test accuracy with image texture features was 0.83 and 0.76, respectively. The highest test accuracy and AUC of chemotherapy response with AUC_max, *KI67*, and *EZRIN* were estimated to be 0.85 and 0.89, respectively. The highest test accuracy and AUC of metastasis with AUC_max, *KI67*, and *EZRIN* were estimated to be 0.85 and 0.8, respectively. The metastasis prediction accuracy increased by 10% using radiogenomics data.

## 1. Introduction

Pediatric osteosarcoma is well-known as one of the most aggressive cancers [1]. Predictions of metastasis and chemotherapy response have a significant impact on pediatric patient survival because metastasis progresses rapidly, and treatment is difficult after the progression of metastasis in pediatric osteosarcoma [2,3]. Chemotherapy responses and cancer metastasis have a profound relationship with gene expression, and the current state of cancer can be identified and predicted by analyzing changes in gene expression [4,5]. Methods of analyzing gene expression or image texture features have previously been used for the diagnosis of chemotherapy responses and metastasis [6,7,8]. *KI67* is a well-known cancer metastasis marker [9]. It is mainly used to indicate cancer metastasis because it is primarily involved in cell division, an important function of metastasis, and an increase in expression was observed when metastasis was actively progressing and the number of cancer cells increased. *KI67* overexpression has been identified in pediatric osteosarcoma [10]. It has also been used as a marker of chemotherapy response [11]. *EZRIN* is a protein constituting the ERM (*EZRIN*-radixin-moesin) family that exists on the cell surface. *EZRIN* plays many roles, including acting as a signaling tube between metastasis-related cell surface molecules and signaling components. Similar to *KI67*, *EZRIN* has been used as a marker for cancer metastasis and chemotherapy response [12,13]. *EZRIN* expression provides an early survival advantage for cancer cells and plays an important role in the invasion of other tissues in pediatric osteosarcoma [14].

Nuclear medicine images, such as positron emission tomography/computed tomography (PET/CT), have also been used to analyze the results of metastasis and chemotherapy responses [15]. The phenotype of cancerous tissues from images or text image features obtained through image analysis can be used to analyze the results of chemotherapy response or cancer metastasis. The combination of genetic expression analysis and nuclear imaging texture features has been used to analyze pre-chemotherapy or chemotherapy responses and metastasis [16,17]. This can be done by observing the phenotype and postponing the change in a given gene because genetic changes in cells lead to changes in the phenotype. A predictive model was estimated using image texture features obtained by analyzing PET/CT images with machine and deep learning [18,19]. In a recent study, the associations between image texture features from tumor ^18^F-FDG PET/CT image texture features and genetic alterations in patients were identified as lung cancer [20]. The related factors were investigated by analyzing the image texture characteristics of the ^18^F-FDG PET/CT image of the gene phenotype.

Radiogenomics technology has also been used to determine whether cancer metastasizes in liver cancer and to estimate a metastasis prediction model [21]. Radiogenomics studies can reveal the current state of cancer by analyzing genetic expression and image texture features. In addition, it is possible to estimate predictive models using machine or deep learning because numerical analysis results, such as gene expression levels and quantitative image texture features, can be derived. In one study, a prediction model was estimated using the machine learning algorithm with a combination analysis of CT images and genetic expression in breast cancer [22]. In another study, image texture features from CT images and gene expression in pancreatic ductal adenocarcinoma were estimated using a prediction model [23].

In this study, we aimed to estimate a predictive model using *KI67*, *EZRIN*, and ^18^F-FDG PET/CT image texture features in pediatric osteosarcoma, which are expressed in relation to metastasis and chemotherapy response. Machine and deep learning techniques were used to construct various predictive models. The accuracy of each model was compared to evaluate a predictive model suitable for metastasis and chemotherapy responses in pediatric osteosarcoma.

## 2. Materials and Methods

### 2.1. Pediatric Osteosarcoma Patient Data

Data from a total of 52 pediatric osteosarcoma patients consisted of 31 male and 21 female children aged <14 years. All of the patients with osteosarcoma received neoadjuvant chemotherapy over four weeks, which involved a combination of methotrexate (a dose of 8–12 g/m^2^), adriamycin (a dose of 60 mg/m^2^), and cisplatin (a dose of 100 mg/m^2^) at intervals of three weeks. The surgery was performed three weeks after the end of the second neoadjuvant chemotherapy. A total of 21 patients expressed both *EZRIN* and *KI67* (Figure S1). *KI67* expression > 15% was classified as *KI67*-positive and <15% was classified as *KI67*-negative. *EZRIN* expression was classified as *EZRIN*-positive or -negative with no *EZRIN* expression. Cancer tissues were collected from the femur, tibia, humerus, and pelvis. All cancer tissues were classified into 2A, 2B, IIA, and unknown according to the American Joint Committee on Cancer stage classification method. The pathologic subtypes of each cancer tissue were identified as osteoblastic (OB), chondroblastic (CB), or others (Table 1). necrosis of 90% or more tumor region indicated a good histological response, and less than 90% tumor region necrosis indicated a poor histological response [24]. A total of 25 patients showed a good histological response to chemotherapy, whereas the remaining 27 patients had no response. In addition, 37 patients had no metastasis, whereas 15 patients had metastasis. Overall, 18 patients had a good histological chemotherapy response and no metastasis, 19 patients had a poor histological chemotherapy response and no metastasis, seven patients had a good histological chemotherapy response and metastasis, and eight patients had a poor histological chemotherapy response and metastasis.

### 2.2. ^18^F-FDG PET/CT Image Texture Features

**Figure 1:**
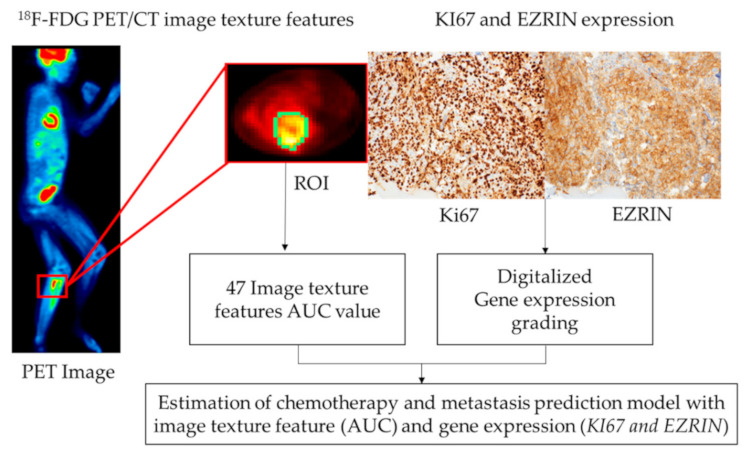
Diagram of the process for generation of the prediction model with image texture features and gene expression.

A total of 52 patient ^18^F-FDG PET/CT images were used for analysis. The ^18^F-FDG PET/CT images were acquired before chemotherapy to confirm the prediction of chemotherapy treatment response in pediatric osteosarcoma patients. Radiomic features were extracted by texture analysis of the acquired ^18^F-FDG PET/CT images. LiFEx (version 4.0) was used for radiomics feature extraction of the ^18^F-FDG PET/CT images. Overall, 47 image texture features were classified as first-order, second-order, and high-order. Figure 1 shows a flow diagram of prediction model generation using image texture features and gene expression.

### 2.3. Feature Selection for the Prediction Model

Among the 47 imaging features, the area under the curve (AUC) values of 0.6 or higher were identified to improve the accuracy of chemotherapy treatment response and metastasis prediction in pediatric osteosarcoma patients. The AUC values of the imaging features were evaluated by analyzing the ^18^F-FDG PET/CT images based on *EZRIN* and *KI67* expression levels. The image texture features for radiogenomics were selected by maximizing the AUC value (AUC_max). Medcalc (version 19.4.1) was used to determine the AUC value of each image feature obtained by extracting the features of the ^18^F-FDG PET/CT images.

### 2.4. Prediction Model Development Using Machine and Deep Learning

Random forest and gradient boosting algorithms were used to predict the treatment response of pediatric osteosarcoma patients. To achieve this goal, the ratio of machine learning training data to test data was set to 7:3. However, owing to the lack of patient datasets, it is difficult to consider any input pre-processing involving the deletion of some data. Cross-validation was performed 10 times to increase the statistical reliability of the performance measurements. A convolutional neural network (CNN; Keras 2.3.1) was used to calculate the accuracy of the prediction model. The CNN consisted of an input layer, an output layer, two convolution layers, two pooling layers, and three fully connected layers. Maximum pooling was used to conserve each layer’s properties. A fully connected layer was used to flatten the two-dimensional layer to a one-dimensional layer. A feature map was extracted from the output layer of the deep learning results. The feature map data were classified as 0 or 1 for the t-distributed stochastic neighbor embedding (t-SNE) plot.

### 2.5. Radiogenomics Data Analysis

Machine learning was performed to evaluate the predictive model for chemotherapy response and metastasis. For the chemotherapy response prediction model, *EZRIN*, *KI67*, image texture features (AUC > 0.6, 7 features) + *EZRIN* + *KI67,* and AUC_max + *EZRIN* + *KI67* were used as inputs. For the metastasis prediction model, *EZRIN*, *KI67*, image texture features (AUC > 0.6, 17 features) + *EZRIN* + *KI67,* and AUC_max + *EZRIN* + *KI67* were used as inputs.

## 3. Results

### 3.1. Image Texture Feature Extraction from ^18^F-FDG PET/CT Images

**Figure 2:**
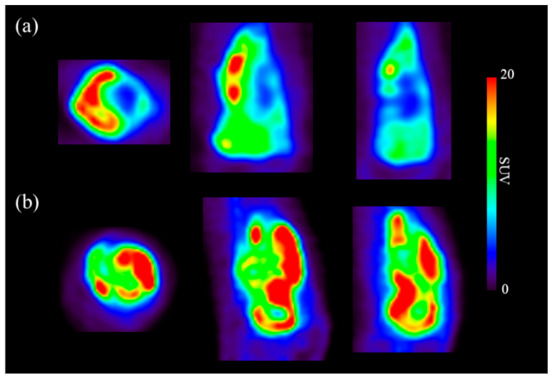
Transverse, coronal, and sagittal sections from osteosarcoma ^18^F-FDG PET/CT images. (**a**) Images from a chemotherapy responder; (**b**) images from a chemotherapy non-responder.

**Figure 3:**
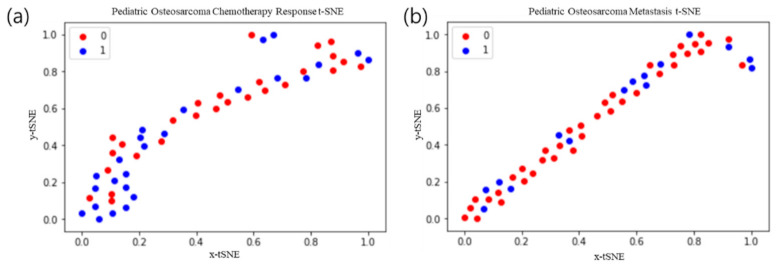
t-Distributed stochastic neighbor embedding (t-SNE) of texture features from 47 images. The 0/1 values represent non-responders and respondents, respectively. (**a**) Chemotherapy response t-SNE; (**b**) metastasis t-SNE.

A total of 47 imaging features (Table S1) were acquired by drawing the region of interest of the tumor site on each ^18^F-FDG PET/CT image from responders/non-responders to chemotherapy and metastasis (Figure 2). Seven of the 47 imaging features had an AUC value of 0.6 or higher for evaluating the chemotherapy response (Table S2). 17 of the 47 imaging features had an AUC value of 0.6 or higher for evaluating the metastasis (Table S3). The image feature with the highest AUC was Neighborhood Gray-Level Different Matrix (NGLDM)_Contrast, for which the value was 0.652 (Table S2). After dimension reduction with t-SNE, the texture features of 47 images from the chemotherapy response and metastasis prediction models did not allow a clear separation of each image (Figure 3).

### 3.2. Machine and Deep Learning Algorithms Using ^18^F-FDG PET/CT Images

**Figure 4:**
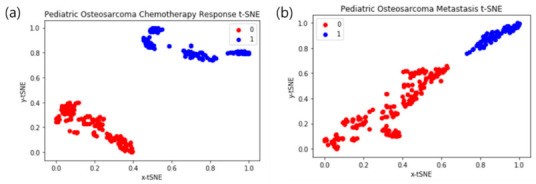
Deep learning t-distributed stochastic neighbor embedding (t-SNE) results. The 0/1 values represent non-responders and respondents, respectively. (**a**) Chemotherapy response t-SNE; (**b**) metastasis t-SNE.

The sensitivity, specificity, AUC, train accuracy, and test accuracy of the prediction models for chemotherapy response and metastasis were calculated using the random forest algorithm, gradient boosting algorithm, and deep learning. The random forest algorithm prediction model test accuracy using total text features (47) and text features (AUC > 0.6) was 0.71 and 0.83 for chemotherapy response, respectively. In the gradient boosting prediction model, the test accuracy using total text features (47) and text features (AUC > 0.6) were 0.81 and 0.81, respectively (Table 2). In the deep learning prediction model, the test accuracy was 0.975 (Figure 4). The accuracy and loss function of chemotherapy response and metastasis were represented in Figure S2. The random forest algorithm prediction model test accuracy using total text features (47) and text features (AUC > 0.6) was 0.72 and 0.76 for metastasis, respectively. In the gradient boosting prediction model, the test accuracy using total text features (47) and text features (AUC > 0.6) was 0.61 and 0.76, respectively. In the deep learning prediction model, the test accuracy was 0.983 (Figure 4). Thus, the prediction models using the random forest algorithm and deep learning showed the highest accuracy for chemotherapy response and metastasis (Table 2).

### 3.3. Deep Learning Interpretation: t-SNE Plots

As shown in Figure 4, after dimension reduction with t-SNE, image texture features from the chemotherapy response and metastasis prediction models were separated into two classes. In the two cases, the classes were clearly separated. We obtained a relatively high precision rate for the chemotherapy response and metastasis prediction model class because the chemotherapy response and metastasis clusters were pure.

### 3.4. Radiogenomics Machine Learning Model

The random forest algorithm was confirmed to be used as a prediction model for the chemotherapy response and metastasis of pediatric osteosarcoma with a combination of gene expression data and image features. The sensitivity, specificity, AUC, train accuracy, and test accuracy of the prediction model were calculated.

The chemotherapy response prediction model AUCs using *EZRIN*, *KI67*, image texture features (7, AUC > 0.6) + *EZRIN* + *KI67,* and NGLDM_Contrast (AUC_max) + *EZRIN* + *KI67* were 0.58, 0.57, 0.77, and 0.89, respectively. The accuracy of the chemotherapy response prediction model using *EZRIN*, *KI67*, image texture features + *EZRIN* + *KI67*, and NGLDM_Contrast (AUC_max) + *EZRIN* + *KI67* was 0.53, 0.52, 0.73, and 0.85, respectively. The metastasis prediction model AUCs using *EZRIN*, *KI67*, image texture features (17, AUC > 0.6) + *EZRIN* + *KI67*, and Gray-Level Co-occurrence Matrix (GLCM)_Correlation (AUC_max) + *EZRIN* + *KI67* were 0.56, 0.57, 0.76, and 0.80, respectively. The metastasis prediction model test accuracy using *EZRIN*, *KI67*, image texture features + *EZRIN* + *KI67*, and GLCM_Correlation (AUC_max) + *EZRIN* + *KI67* was 0.54, 0.52, 0.74, and 0.85, respectively. The prediction model using AUC_max, *EZRIN*, and *KI67* with the random forest algorithm showed the highest accuracy (Table 3).

### 3.5. Machine Learning Prediction Model with the Random Forest Algorithm

**Figure 5:**
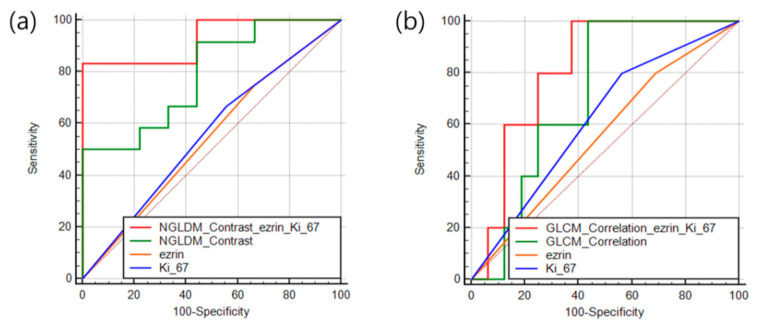
Receiver operating characteristic curves of the prediction features for patient outcomes. (**a**) Chemotherapy response. (**b**) Metastasis.

The receiver operating characteristic curves of the chemotherapy response and metastasis prediction models are shown in Figure 4. The AUCs for chemotherapy prediction using *KI67*, *EZRIN*, image texture features + *EZRIN* + *KI67*, and NGLDM_Contrast + *EZRIN* + *KI67* were 0.58, 0.57, 0.77, and 0.89, respectively. The AUCs for metastasis prediction using *KI67*, *EZRIN*, image texture features + *EZRIN* + *KI67*, and GLCM_Correlation + *EZRIN* + *KI67* were 0.56, 0.57, 0.76, and 0.8, respectively (Figure 5).

## 4. Discussion

In this study, we evaluated a predictive model that can predict the chemotherapy response and metastasis of pediatric osteosarcoma by analyzing gene expression and ^18^F-FDG PET/CT image texture features. Several appropriate algorithms were selected from machine learning algorithms that have shown good predictive performance. Imaging features that are associated with metastasis and chemotherapy response were extracted. A predictive model showing high accuracy was estimated using the extracted image features, gene expression, and the previously selected algorithm.

*KI67* and *EZRIN* are clinically used as biomarkers to determine metastasis or chemotherapy responses [25,26,27,28]. Rejniak et al. reported that *KI67* expression is associated with pediatric osteosarcoma metastasis and chemotherapy response [29], and Bacci et al. reported that *EZRIN* expression is associated with pediatric osteosarcoma metastasis and chemotherapy response [30]. In other studies, it is well-known that the expression levels of *KI67* and *EZRIN* are associated with metastasis and chemotherapy responses in pediatric osteosarcoma [31,32,33,34,35,36]. As described previously, these two genes were previously used as biomarkers for pediatric osteosarcoma, but the prediction model test accuracy was low in our study (test accuracy ~ 0.53). Low accuracy was estimated despite the use of well-known biomarkers for chemotherapy response and metastasis. The predictive model of chemotherapy response and metastasis suggests that accurate prediction is difficult using the expression of a single gene.

A total of 47 image features were extracted from the ^18^F-FDG PET/CT images. Imaging features related to chemotherapy response and metastasis were classified by the AUC value. The image texture feature that was most closely related to chemotherapy response was NGLDM_Contrast, and related to metastasis was GLCM_Correlation. The prediction model with AUC_max showed low accuracy in chemotherapy response and metastasis. The chemotherapy results showed a high predictive accuracy than metastasis from the prediction models estimated using image texture features.

The predictive model using imaging texture features showed an accuracy of 83% for chemotherapy response and 76% for metastasis. For clinical applications, it is necessary to generate a predictive model with higher accuracy. We used a radiogenomics technique that evaluates both gene expression and imaging factors to improve the accuracy of the predictive model for both conditions. The predictive model using the radiogenomic technique showed high predictive ability in both chemotherapy response and metastasis (Figure 4). The accuracy was improved by about 10% or more when the AUC_max value was used in both conditions.

The predictive model using images to predict chemotherapy responses showed good results. In metastasis, the predictive model that used images and genetic information displayed improved performance. Deep learning has shown high predictive performance with image texture features, but it is difficult to apply genetic information with image texture features to improve accuracy. Machine learning predictive models can be applied to data with a variety of properties, such as gene expression and image texture features, as in this study. Additionally, factors other than gene expression and image factors can be used when conducting research on machine learning predictive models with digitalization of data.

The predictive model of chemotherapy using machine learning showed a high accuracy of 83% when estimated by ^18^F-FDG PET/CT imaging alone and 85% when analyzed after adding gene expression. The chemotherapy response reaction process is related to the heterogeneity of cancer cells, and since this can be confirmed by imaging, it is possible to obtain higher accuracy with the analysis results of the image. It is difficult to obtain high accuracy through image analysis alone because of the challenge in determining the metastasis process through simple image analysis. The accuracy of the metastasis process was 76%, and when the genetic analysis results were added, it increased to 85%. A more accurate predictive model was estimated by adding the gene expression results to the image analysis of the metastasis process.

A limitation of this study is that owing to the rarity of pediatric osteosarcoma, a patient set for extra validation could not be obtained. The predictive model was generated using the radiogenomics technique, but the accuracy of the predictive model was not high even in other patient groups through additional verification. A larger population of pediatric osteosarcoma patients is needed to evaluate the accuracy of the predictive model. The data in this study may not be reliable because of the small sample size. However, it can be used as preliminary evidence to estimate the probability of the predictive model. Even though the number of patient groups is small, the analysis method using image texture features and gene expression data have been shown to be applicable to chemotherapy and metastasis prediction models. Additional data from other pediatric patients with osteosarcoma could improve the accuracy of the model for predicting chemotherapy response and metastasis.

## 5. Conclusions

Predictive models using the random forest algorithm showed the best accuracy for predicting metastasis and chemotherapy responses in our pediatric osteosarcoma dataset. The predictive model that combined *KI67*, *EZRIN*, and image texture features was estimated to have a higher accuracy than the predictive models using each factor separately. The accuracy of metastasis prediction increased by 10% using radiogenomics data. High accuracy was estimated using a radiogenomics technique that uses both gene expression and imaging texture features for metastasis prediction. Thus, a predictive model using radiogenomics technology that incorporates both imaging features and gene expression can accurately predict metastasis and chemotherapy responses to improve patient outcomes.

## Figures and Tables

**Table 1:** Patient information.

Characteristic	Value
Sex, *n* (%)	21 (40.38%)
Female	31 (59.61%)
Male	
Age, *n* (%)	52 (100%)
Years ≤ 14	
Location of primary tumor, *n* (%)	33 (63.46%)
Femur	16 (30.76%)
Tibia	2 (3.84%)
Humerus	1 (1.92%)
Pelvis	
AJCC stage, *n* (%)	13 (25%)
2A	16 (30.76%)
2B	4 (7.69%)
IIA	19 (36.53%)
Unknown	
Pathologic subtype, *n* (%)	39 (75%)
OB (Osteoblastic)	10 (19.23%)
CB (Chondroblastic)	3 (5.76%)
Others	

**Table 2:** Chemotherapy response and metastasis prediction model with various machine learning algorithms and deep learning.

**Chemotherapy Response**	**Random Forest**		**Gradient Boosting**		**Deep Learning**
	**Text Feature (47)**	**AUC > 0.6 (7)**	**Text Feature (47)**	**AUC > 0.6 (7)**	**Train (37): Test (15)**
Sensitivity	0.76	0.79	0.85	0.84	0.956
Specificity	0.74	0.82	0.94	0.88	0.964
AUC	0.76	0.80	0.88	0.86	0.917
Train accuracy	0.71	0.83	0.77	0.83	0.978
Test accuracy	0.71	0.83	0.81	0.81	0.975
**Metastasis**	**Random Forest**		**Gradient Boosting**		**Deep Learning**
	**Text Feature (47)**	**AUC > 0.6 (17)**	**Text Feature (47)**	**AUC > 0.6 (17)**	**Train (37): Test (15)**
Sensitivity	0.77	0.80	0.76	0.85	0.958
Specificity	0.74	0.66	0.76	0.73	0.990
AUC	0.73	0.85	0.74	0.72	0.970
Train accuracy	0.72	0.76	0.77	0.67	0.986
Test accuracy	0.72	0.76	0.61	0.76	0.983

**Table 3:** Chemotherapy Response and metastasis prediction model with gene expressions, combination of gene expression image texture features and gene expression and combination of area under curve max (AUC_max) image texture and gene expressions.

**Chemotherapy Response**	***EZRIN***	***KI67***	**Image Texture Feature + *EZRIN* + *KI67***	**NGLDM_Contrast + *EZRIN*+ *KI67***
Sensitivity	0.59	0.57	0.84	0.87
Specificity	0.44	0.68	0.75	0.85
AUC	0.58	0.57	0.77	0.89
Train accuracy	0.53	0.52	0.73	0.85
Test accuracy	0.53	0.52	0.73	0.85
**Metastasis**	***EZRIN***	***KI67***	**Image Texture Feature** **+ *EZRIN* + *KI67***	**GLCM_Correlation + *EZRIN*+ *KI67***
Sensitivity	0.61	0.54	0.77	0.91
Specificity	0.42	0.65	0.55	0.6
AUC	0.56	0.57	0.76	0.8
Train accuracy	0.54	0.52	0.74	0.85
Test accuracy	0.54	0.52	0.74	0.85

## Data Availability

Data sharing is not applicable to this article.

## Supplementary Materials

The following are available online at https://www.mdpi.com/article/10.3390/cancers13112671/s1, Figure S1. Immunohistochemical staining of KI67 (a) and EZRIN (b). Figure S2. ^18^F-FDG PET/CT image deep learning accuracy and loss value, (a) Chemotherapy response prediction, (b) Metastasis prediction. Table S1. The AUC values of image texture features for chemotherapy response from 52 pediatric osteosarcoma. Table S2. The AUC values of image texture features for metastasis from 52 pediatric osteosarcoma. Table S3. 47 image texture features from ^18^F-FDG PET/CT.
