# Supplementary material for: Preliminary Radiogenomic Evidence for the Prediction of Metastasis and Chemotherapy Response in Pediatric Patients with Osteosarcoma Using 18F-FDG PET/CT, EZRIN, and KI67
Source: Cancers (Basel). 2021 May 28;13(11):2671. doi: 10.3390/cancers13112671 (PMC8198322; doi:10.3390/cancers13112671)
Supplement: Supplementary file 1 [file cancers-13-02671-s001.zip › cancers-1232486-supple-final.pdf]

Supplementary Materials

Preliminary Radiogenomic Evidence for the Prediction of Metastasis and Chemotherapy Response in Pediatric Patients with Osteosarcoma Using ^{18}F-FDG PET/CT, *EZRIN*, and *KI67*

Byung-Chul Kim, Jingyu Kim, Kangsan Kim, Byung Hyun Byun, Ilhan Lim, Chang-Bae Kong, Won Seok Song, Jae-Soo Koh and Sang-Keun Woo

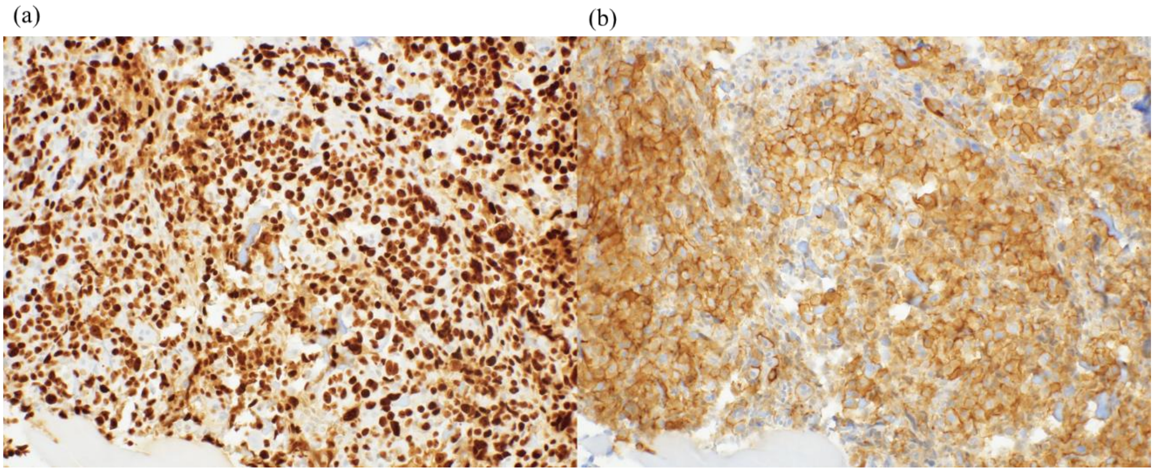

Figure S1. Immunohistochemical staining of *KI67* (**a**) and *EZRIN* (**b**).

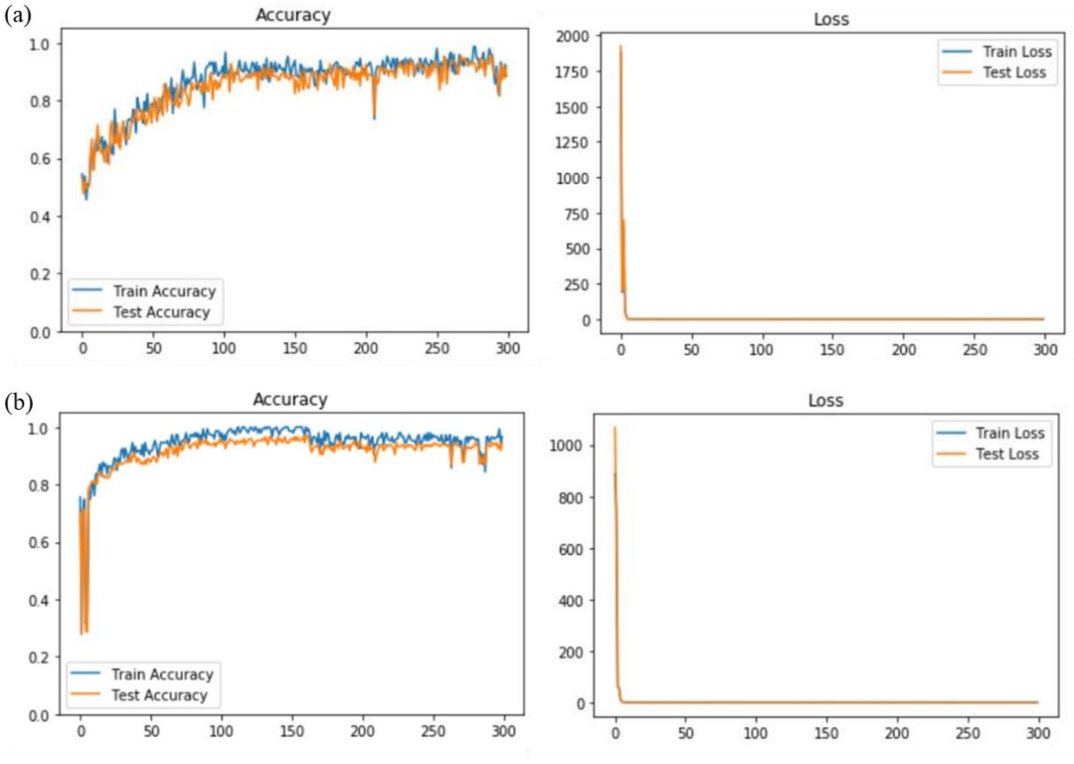

Figure S2. ^{18}F-FDG PET/CT image deep learning accuracy and loss value, (**a**) Chemotherapy response prediction, (**b**) Metastasis prediction.

Table S1. The AUC values of image texture features for chemotherapy response form 52 pediatric osteosarcoma.

Feature	AUC
“NGLDM_Contrast”	0.652
“HISTO_Kurtosis”	0.647
“GLZLM_LZHGE”	0.644
"CONVENTIONAL_SUVmin (SUV)"	0.616
“GLRLM_RP”	0.609
“GLCM_Homogeneity”	0.604
“GLRLM_SRE”	0.604

Table S2. The AUC values of image texture features for metastasis form 52 pediatric osteosarcoma.

Feature	AUC
“GLZLM_SZE”	0.68
“HISTO_Entropy_log2”	0.624
“HISTO_Energy”	0.624
“HISTO_Entropy_log10”	0.623
"CONVENTIONAL_TLG (mL) (value only for PET or NM)"	0.622
“GLZLM_SZLGE”	0.622
“GLZLM_ZLNU”	0.62
“SHAPE_Compacity”	0.619
"CONVENTIONAL_SUVmin (SUV)"	0.618
“GLCM_Entropy_log2”	0.617
“GLCM_Entropy_log10”	0.616
“HISTO_Skewness”	0.613
“GLZLM_GLNU”	0.608
“GLCM_Energy”	0.607
“SHAPE_Sphericity”	0.603
“SHAPE_Volume_No_vx”	0.6
“GLRLM_RLNU”	0.6

Table S3. 47 image texture features from ^{18}F-FDG PET/CT.

image texture features(IBSI)	AUC
SUVmax	0.424
SUVmean	0.453
SUVmin	0.64
SUVpeak	0.436
SUV_SD	0.4
TLG	0.362
Gray-Level Co-occurrence Matrix_Contrast(GLCM_contrast)	0.453
Gray-Level Co-occurrence Matrix_Correlation(GLCM_Correlation)	0.526
Gray-Level Co-occurrence Matrix_DEPRECATED Dissimilarity(GLCM_DEPRECATED Dissimilarity)	0.553
Gray-Level Co-occurrence Matrix_Joint Energy(GLCM_Joint Energy)	0.494
Gray-Level Co-occurrence Matrix_Joint Entropy (GLCM_Joint Entropy)	0.54
Gray-Level Co-occurrence Matrix_Difference Entropy(GLCM_Difference Entropy)	0.54
Gray-Level Co-occurrence Matrix_DEPRECATED. Homogeneity 1(GLCM_DEPRECATED. Homogeneity 1)	0.5
Gray Level Run Length Matrix_Gray Level Non-Uniformity (GLRLM_GLN)	0.507
Gray Level Run Length Matrix_High Gray Level Run Emphasis (GLRLM_HGLRE)	0.478
Gray Level Run Length Matrix_Low Gray Level Run Emphasis (GLRLM_LGLRE)	0.568
Gray Level Run Length Matrix_Long Run Emphasis (GLRLM_LRE)	0.499
Gray Level Run Length Matrix_Long Run High Gray Level Emphasis (GLRLM_LRHGLE)	0.482
Gray Level Run Length Matrix_Long Run LOW Gray Level Emphasis (GLRLM_LRLGLE)	0.557
Gray Level Run Length Matrix_Run Length Non-Uniformity (GLRLM_RLN)	0.465
Gray Level Run Length Matrix_Run Percentage (GLRLM_RP)	0.568
Gray Level Run Length Matrix_Short Run Emphasis (GLRLM_SRE)	0.56
Gray Level Run Length Matrix_Short Run High Gray Level Emphasis (GLRLM_SRHGLE)	0.482
Gray Level Run Length Matrix_Short Run LOW Gray Level Emphasis (GLRLM_SRLGLE)	0.565
Gray Level Size Zone Matrix_Gray Level Non-Uniformity (GLSZM_GLN)	0.524

Gray Level Size Zone Matrix_High Gray Level Zone Emphasis (GLSZM_HGLZE)	0.454
Gray Level Size Zone Matrix_Low Gray Level Zone Emphasis (GLSZM_LGLZE)	0.551
Gray Level Size Zone Matrix_Large Area Emphasis (GLSZM_LAE)	0.491
Gray Level Size Zone Matrix_Large Area High Gray Level Emphasis (GLSZM_LAHGLE)	0.55
Gray Level Size Zone Matrix_Large Area Low Gray Level Emphasis (GLSZM_LALGLE)	0.521
Gray Level Size Zone Matrix_Small Area Emphasis (GLSZM_SAE)	0.546
Gray Level Size Zone Matrix_Small Area High Gray Level Emphasis (GLSZM_SAHGLE)	0.453
Gray Level Size Zone Matrix_Small Area Low Gray Level Emphasis (GLSZM_SALGLE)	0.596
Gray Level Size Zone Matrix_Size-Zone Non-Uniformity (GLSZM_SZN)	0.478
Gray Level Size Zone Matrix_Zone Variance (GLSZM_ZV)	0.541
entropy	0.549
entropy_log10	0.547
entropy_log2	0.547
Kurtosis	0.471
Skewness	0.478
Neighbouring Gray Tone Difference Matrix_Busyness (NGTDM_Busyness)	0.474
Neighbouring Gray Tone Difference Matrix_Coarseness (NGTDM_Coarseness)	0.469
Neighbouring Gray Tone Difference Matrix_Contrast (NGTDM_Contrast)	0.568
XSpatialResampling	0.617
YSpatialResampling	0.617
ZSpatialResampling	0.546
